# Impact of body mass index on aortic valve stenosis and its clinical outcomes in early adulthood: a long-term study of young men

**DOI:** 10.1038/s41598-026-44041-2

**Published:** 2026-03-26

**Authors:** Martin Lindgren, Silvana Kontogeorgos, Amir Djekic, Martin Adiels, Susanne J. Nielsen, Josefina Robertson, Zacharias Mandalenakis, Maria Åberg, N. David Åberg, Demir Djekic

**Affiliations:** 1 Department of Molecular and Clinical Medicine, Institute of Medicine, Sahlgrenska Academy, University of Gothenburg, Gothenburg, Sweden; 2 Department of Medicine, Geriatrics and Emergency Medicine, Sahlgrenska University Hospital, Östra Hospital, Region Västra Götaland, Gothenburg, Sweden; 3 School of Public Health and Community Medicine/Primary Health Care, Institute of Medicine, Sahlgrenska Academy, University of Gothenburg, Gothenburg, Sweden; 4 Department of Infectious Diseases, Region Västra Götaland, Sahlgrenska University Hospital, Gothenburg, Sweden; 5 Region Västra Götaland, Regionhälsan, Gothenburg, Sweden; 6 Department of Internal Medicine, Institute of Medicine, The Sahlgrenska Academy, University of Gothenburg, Gothenburg, Sweden; 7 Department of Acute Medicine and Geriatrics (SU/Sahlgrenska), Region Västra Götaland, Sahlgrenska University Hospital, Gothenburg, Sweden; 8 Department of Cardiology, Sahlgrenska University Hospital/Östra, Diagnosvägen 11, 416 85 Gothenburg, Sweden

**Keywords:** Obesity, Body mass index, Aortic valve stenosis, Mortality, Heart failure, Cardiology, Diseases, Health care, Medical research, Risk factors

## Abstract

We investigated associations between body mass index (BMI) in adolescence with development of aortic valve stenosis (AVS) in early adulthood, and clinical outcomes among those who develop AVS. We used data from 1 701 390 men (mean age, 18.3 ± 0.8 years) enrolled in compulsory conscription for military service in Sweden from 1969 to 2005. Anthropometrics, blood pressure, fitness, muscle strength and IQ were recorded at baseline. Over a median follow-up of 32 years (IQR, 24–41 years), 5766 men were diagnosed with AVS (mean age 54.7 ± 9.4 years). After multivariable adjustment, individuals with a BMI of < 18.5 kg/m^2^, compared to those with a low-normal BMI (20.0–<22.5 kg/m^2^) showed lower risk (HR 0.70; 95% CI 0.61–0.80) of AVS. Higher BMI categories were progressively associated with increased risk, reaching HR 1.91 (95% CI 1.12–3.25) for BMI 35–50 kg/m². Further on, those diagnosed with AVS were followed for a median of ~ 5.5 years. The multivariable-adjusted HR for all-cause mortality, cardiovascular mortality, and heart failure for obese (BMI 30–50 kg/m^2^), compared to BMI < 20.0 kg/m^2^ in men diagnosed with AVS, were 4.69 (95% CI 2.61–8.44), 4.09 (95% CI 1.72–9.73), and 2.76 (95% CI, 1.36–5.60), respectively. Higher BMI in youth, even within the conventional normal range, is associated with an elevated risk of developing AVS in early adulthood, whereas low BMI appears protective. Long-term exposure of obesity in individuals with AVS is associated with worse survival and higher risk of cardiovascular events.

**Supplementary Information:**

The online version contains supplementary material available at 10.1038/s41598-026-44041-2.

## Introduction

Obesity is defined as an excessive accumulation of body fat, and is associated with numerous disorders, including cardiovascular disease^1–3^. In 2016, it was estimated that more than 1.9 billion adults, equivalent to 39% of the world’s adult population, were overweight, and 650 million (12%) were obese^4^. Globally, the prevalence of obesity has approximately tripled since 1975, and it is anticipated that by 2030, 33 of 53 European countries will reach an obesity prevalence exceeding 30%, with rising rates also among children and adolescents^4–6^. In parallel, with rising life expectancy, the global proportion of those aged > 65 years will increase to an anticipated 15% in 2040 from 5% in 1975^7^. Overweight or high normal weight is frequently established already in childhood or early adulthood, but little is known about how these lower ranges may affect risk profiles for many cardiovascular diseases. Because rates of cardiovascular disease rise markedly with age, the long-term consequences of early life obesity on cardiovascular disease need to be investigated from a life course perspective.

Aortic valve stenosis (AVS) is the most common heart valve disease in high-income countries^8,9^. Indeed, prospective studies have found risk factors associated with AVS to be similar to those for atherosclerotic cardiovascular disease: age, smoking, hypertension, diabetes mellitus, elevated cholesterol levels, and obesity^10–13^. Despite this, few studies have investigated the long-term association of traditional atherosclerosis risk factors, including obesity, with AVS development. Obesity in middle age is associated with a two-fold increase of development of AVS^14^. However, information about the incidence of AVS among individuals at the extremes of the BMI distribution (< 18.5 and > 40 kg/m^2^) and among those with elevated BMI in youth is lacking. Furthermore, it is not known whether obesity early in life has an impact on well-known clinical outcomes of established AVS such as all-cause mortality, heart failure (HF), cardiac arrhythmias, acute myocardial infarction (AMI), and ischemic stroke (IS).

We conducted a long-term prospective cohort study using data from military conscripts to investigate the relationship between BMI in youth and subsequent diagnosis of AVS. A second aim was to determine the association between obesity and clinical outcomes in individuals diagnosed with AVS.

## Methods

### Study population

**Fig. 1:**
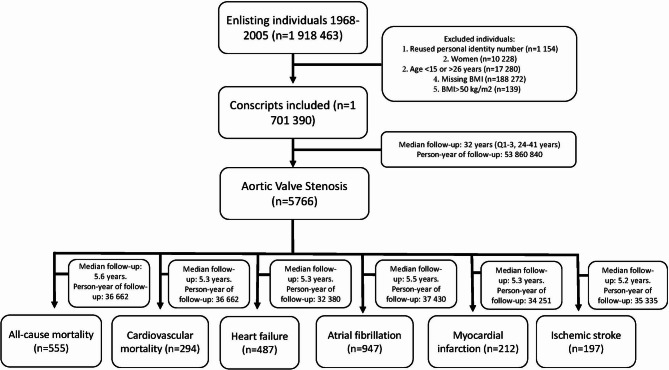
Study flowchart.

This was a prospective cohort study in which data were drawn from the Swedish Military Service Conscription Register (*n* = 1 918 463), including inductees into military service between 1969 and 2005 (Fig. 1). Conscription for military service was compulsory until 2005, except for prison inmates and those with a severe medical condition or functional disabilities (~ 2–3% annually). Women were excluded from the study (*n* = 10 228), as well as men < 15 and > 26 years of age at conscription (*n* = 17 280) and those with missing height or weight data (*n* = 188 272).

All military conscripts underwent a two-day assessment at one of six centres in Sweden. The protocols were conducted according to standard procedures and included measurements of weight, height, and blood pressure, as well as a review of medical history. Cognitive evaluations provided a global intelligence score (intelligence quotient, IQ) as a measure of general cognitive ability^15,16^. The test results were standardized and transformed to Standard Nine (stanine) scores 1 to 9. Cardiorespiratory fitness was determined using a bicycle ergometric test, and the final work rate (Wmax) divided by body weight was recorded. For the conscription years 1972 to 2005, raw data of Wmax per kilogram were available. From 1969 to 1971 raw data were not recorded, but the values of Wmax/kg were converted to nine levels that served as a measure of fitness. No raw data were recorded for the conscripts performing at the lowest three fitness levels during 2000 through 2005. During this period, these levels were handled as missing in the analysis. Isometric muscle strength was measured by hand grip, elbow flexion, and knee extension. Weighted values were integrated into an overall estimate in kiloponds until April 1, 1979, and in Newtons after that date. Test results were standardized against data from previous years, resulting in scores from 1 to 9 (low to high). The longitudinal integration database for health insurance and labour market (LISA) was used to obtain data about parental education (80% coverage) and is categorized as < 9 years, pre-high school education of 9 years, high school education, < 2 years university, ≥ 2 years university, postgraduate education, and postgraduate research training.

### Outcomes

The Swedish universal healthcare system offers hospital inpatient and outpatient care at low cost to all citizens. Data from visits or hospitalizations are recorded in the Swedish National Hospital Register (NPR) with an increased coverage from 1970 to 1986. NPR has a complete national coverage from 1987 for all diagnoses and all hospitalized patients and from 2001, from all outpatient visits. AVS cases and cardiovascular outcomes were identified according to the International Classification of Diseases (ICD)−10 and linked to the Hospital Register and Cause of Death Register through the Swedish 12-digit personal identification number (Table S1)^17–19^.

### Statistical analysis

Subjects were followed up from date of conscription until an initial AVS diagnosis, death, emigration, or to December 31, 2019, whichever occurred first. The start of subsequent follow-up for those diagnosed with AVS was at the date of diagnosis. We applied traditional statistical methods to assess the strength of the association between BMI in adolescence and development of AVS and for clinical outcomes in the subcohort who had developed AVS with additional years of follow-up. Poisson regression was used to calculate incidence rates and their corresponding 95% confidence intervals (CI). Cox proportional hazard models were used to estimate the association between adolescent BMI and future hospitalization for AVS. BMI, calculated as weight in kg divided by height in m squared (kg/m^2^), was categorized as < 18.5, 18.5–<20, 20–<22.5, 22.5–<25, 25–<27.5, 27.5 < 30, 30–<35, and 35–<50 kg/m^2^. The BMI reference was set as 20–<22.5 kg/m^2^.

Those who developed AVS formed a subcohort that was followed until a first clinical cardiovascular outcome of either death, cardiovascular mortality, HF, atrial fibrillation, AMI, or IS. Incidence rates and their corresponding 95% confidence intervals (CI) for clinical events were calculated with Poisson regression. Cox proportional hazard models were used to estimate the association between adolescent BMI and future hospitalization for clinical outcomes in adulthood. BMI was categorized in fewer categories; <20, 20–<25.0, 25.0–<30.0, and 30–50 kg/m^2^, since relatively few clinical outcomes developed during the study follow-up (BMI reference was here defined as < 20.0 kg/m^2^). The unadjusted models are presented in Supplemental Material (Figure S1-S7).

Blood pressure was categorized as systolic of 100–119, 120–125, 126–130, 131–138, and 139–180 mmHg and diastolic of 40–59, 60–65, 66–70, 71–76, and 77–100 mmHg. Cardiorespiratory fitness was classified as low (1–4), moderate (5–7), or high (8 and 9) and muscle strength as low (1–3), moderate (4–6), or high (7–9). Parental education was categorized as low (pre–high school, ≤ 9 years), moderate (high school, 10–12 years), or high (university and higher, > 12 years). Differences among induction centres, conscription years, and age at conscription were adjusted for as potential confounders. Spline plots were produced with BMI as a restricted cubic spline with degree 3 and 4 knots placed at 5% (18.0 kg/m^2^), 35% (20.5 kg/m^2^), 65% (22.4 kg/m^2^), and 95% (27.5 kg/m^2^). Year of conscription was included in the spline with knots placed at 5% (1971), 35% (1982), 65% (1992), and 95% (2004). Interactions of BMI with other variables were tested with BMI as a continuous variable. Systolic and diastolic blood pressure, cardiorespiratory fitness, muscle strength, baseline diabetes mellitus, hypertension, and congenital heart disease were analysed as factors. The Ethics Committee of the University of Gothenburg approved the study (Dnr 567 − 15), which conformed to the principles outlined in the Declaration of Helsinki. The requirement for informed consent was waived by the Ethics Committee because the study was based on anonymized historical registry data, for which consent is not required under Swedish national regulations. Data were prepared with SAS version 9.4 (SAS Institute, Cary, NC), and statistical analyses were conducted using R software version 3.4.2 (moonBook, ggplot, caret, rms, survival packages) (R Foundation for Statistical Computing, Vienna, Austria). The datasets used in this study contain sensitive personal information from national registries and cannot be made publicly available under Swedish data protection regulations. However, anonymized data may be available from the corresponding author upon reasonable request.

## Results

### Study population characteristics

After exclusions, 1 701 390 subjects were included in the study (mean age, 18.3 ± 0.8 years, Fig. 1). Descriptive data regarding BMI categories and other index parameters are presented in Table 1. Of the total study cohort, 8.1% were underweight (BMI < 18.5 kg/m^2^), 79.5% normal (BMI, 18.5–<25 kg/m^2^), 10.0% overweight (BMI, 25–<30 kg/m^2^), and 2.2% were obese (BMI 30–50 kg/m^2^). Systolic and diastolic blood pressure increased with higher BMI. Cardiorespiratory fitness was lower in men with BMI > 25 kg/m^2^ compared with those with BMI 20.0–<22.5 kg/m^2^ and was lowest in those with BMI 35–50 kg/m^2^. Similar trends were observed for IQ and parental education. There were no major differences in diagnoses of adult congenital heart disease at baseline among BMI groups. Diagnoses of hypertension at baseline increased with higher BMI (Table 1).

**Table 1:** Baseline characteristics of study population relative to body mass index (BMI kg/m^2^) at conscription.

	All	BMI < 18.5 kg/m^2^	BMI 18.5–<20.0 kg/m^2^	BMI 20.0–<22.5 kg/m^2^	BMI 22.5–<25.0 kg/m^2^	BMI 25.0–<27.5 kg/m^2^	BMI 27.5–<30.0 kg/m^2^	BMI 30.0–<35.0 kg/m^2^	BMI 35.0– 50 kg/m^2^
Men, *n* (% of total)	1,701,390(100)	138,550 (8.1)	306,878 (18.0)	686,154 (40.3)	361,514 (21.2)	125,662 (7.4)	44,399 (2.6)	30,701 (1.8)	7532 (0.4)
Age (SD), years	18.3 (0.8)	18.3 (0.6)	18.3 (0.6)	18.3 (0.7)	18.3 (0.7)	18.4 (0.8)	18.4 (0.8)	18.3 (0.8)	18.4 (0.7)
Height (SD), cm	179.2 (6.6)	180.0 (7.0)	179.4 (6.6)	179.3 (6.5)	179.1 (6.6)	178.9 (6.5)	179.0 (6.8)	179.2 (6.8)	179.4 (6.8)
Weight (SD), kg	70.4 (11.17)	57.0 (5.3)	62.2 (4.8)	68.3 (5.4)	75.6 (6.0)	83.5 (6.6)	91.8 (7.4)	102.5 (9.1)	121.8 (12.2)
BMI (SD), kg/m2	21.9 (3.1)	17.7 (1.0)	19.3 (0.4)	21.2 (0.7)	23.6 (0.7)	26.1 (0.7)	28.6 (0.7)	31.9 (1.4)	37.8 (2.6)
Systolic BP (SD), mmHg	128.5 (11.1)	125.4 (11.2)	126.6 (10.9)	128.2 (10.9)	129.9 (10.9)	131.3 (10.9)	132.5 (11.2)	133.9 (11.1)	136.5 (12.1)
Diastolic BP (SD), mmHg	67.6 (9.9)	67.4 (9.8)	67.3 (9.7)	67.4 (9.8)	67.7 (9.9)	68.5 (10.1)	69.3 (10.4)	70.5 (10.6)	72.8 (11.2)
Wmax (SD)	251.6 (96.5)	196.9 (94.6)	225.5 (95.2)	255.1 (94.6)	276.9 (91.6)	279.2 (88.8)	276.1 (84.9)	273.0 (82.8)	266.0 (83.8)
Wmax/kg (SD)	3.6 (1.4)	3.5 (1.7)	3.6 (1.5)	3.7 (1.4)	3.7 (1.2)	3.4 (1.1)	3.0 (0.9)	2.7 (0.8)	2.2 (0.7)
Cardiorespiratory fitness, n (%)									
Low (1–4)	260,517 (15.7)	43,117 (32.4)	53,724 (17.9)	69,527 (10.3)	36,406 (10.3)	23,719 (19.5)	14,904 (35.0)	15,141 (53.9)	3979 (74.0)
Moderate (5–6)	807,458 (48.8)	74,550 (56.1)	172,012 (57.2)	324,210 (48.2)	152,278 (43.2)	56,359 (46.4)	18,326 (43.0)	8820 (31.4)	903 (16.8)
High (7–9)	588,020 (35.5)	15,212 (11.4)	74,898 (24.9)	278,519 (41.4)	163,982 (46.5)	41,466 (34.1)	9340 (21.9)	4106 (14.6)	497 (9.2)
Muscle strength, n (%)									
Low (1–3)	237,863 (14.1)	59,249 (43.6)	67,667 (22.2)	73,805 (10.8)	23,417 (6.5)	8055 (6.5)	3024 (6.9)	2100 (7.1)	546 (8.9)
Moderate (4–6)	953,985 (56.5)	72,674 (53.5)	205,643 (67.3)	420,542 (61.6)	171,804 (47.8)	52,306 (41.9)	17,602 (40.0)	11,192 (37.8)	2222 (36.0)
High (7–9)	49,640 (29.4)	3891 (2.9)	32,057 (10.5)	188,667 (27.6)	164,347 (45.7)	64,418 (51.6)	23,343 (53.1)	16,349 (55.2)	3398 (55.1)
IQ, n (%)									
Low (1–3)	342,167 (20.3)	30,803 (22.5)	60,882 (19.9)	125,565 (18.4)	71,071 (19.8)	29,932 (24.0)	12,400 (28.2)	9136 (30.6)	2378 (35.4)
Moderate (4–6)	923,468 (54.7)	71,236 (52.1)	162,840 (53.4)	373,707 (54.8)	201,516 (56.1)	70,075 (56.2)	24,201 (55.1)	16,376 (54.8)	3517 (52.3)
High (7–9)	423,013 (25.1)	34,706 (25.4)	81,453 (26.7)	183,220 (26.8)	86,528 (24.1)	24,578 (19.7)	7327 (16.7)	4369 (14.6)	832 (12.4)
Parental education, n (%)									
Low (≤ 9 years)	226,656 (16.1)	20,895 (19.0)	43,140 (17.3)	89,925 (15.8)	45,633 (15.1)	16,326 (15.5)	5921 (15.9)	3907 (15.1)	909 (14.1)
Moderate (10–12 years)	870,961 (62.0)	66,516 (60.6)	150,270 (60.4)	345,851 (60.9)	190,586 (62.9)	69,450 (65.8)	25,375 (68.0)	18,202 (70.4)	4711 (73.1)
High (> 12 years)	306,841 (21.8)	22,310 (20.3)	55,353 (22.3)	132,078 (23.3)	66,783 (22.0)	19,695 (18.7)	6046 (16.2)	3751 (14.5)	825 (12.8)
Diagnosis at baseline, n (%)									
ACHD	2493 (0.1)	239 (0.2)	424 (0.1)	947 (0.1)	550 (0.2)	187 (0.1)	76 (0.2)	58 (0.2)	12 (0.2)
Diabetes mellitus	1337 (0.1)	90 (0.1)	189 (0.1)	537 (0.1)	342 (0.1)	115 (0.1)	29 (0.1)	24 (0.1)	11 (0.1)
Hypertension	2797 (0.2)	115 (0.1)	264 (0.1)	858 (0.1)	647 (0.2)	370 (0.3)	228 (0.5)	217 (0.7)	98 (1.3)

Variables are shown as n (%) or mean (SD). BMI indicates body mass index; BP, blood pressure; Wmax, maximum work capacity; IQ, intelligence quotient; and ACHD, Adult congenital heart disease.

### Risk of aortic valve stenosis

**Fig. 2:**
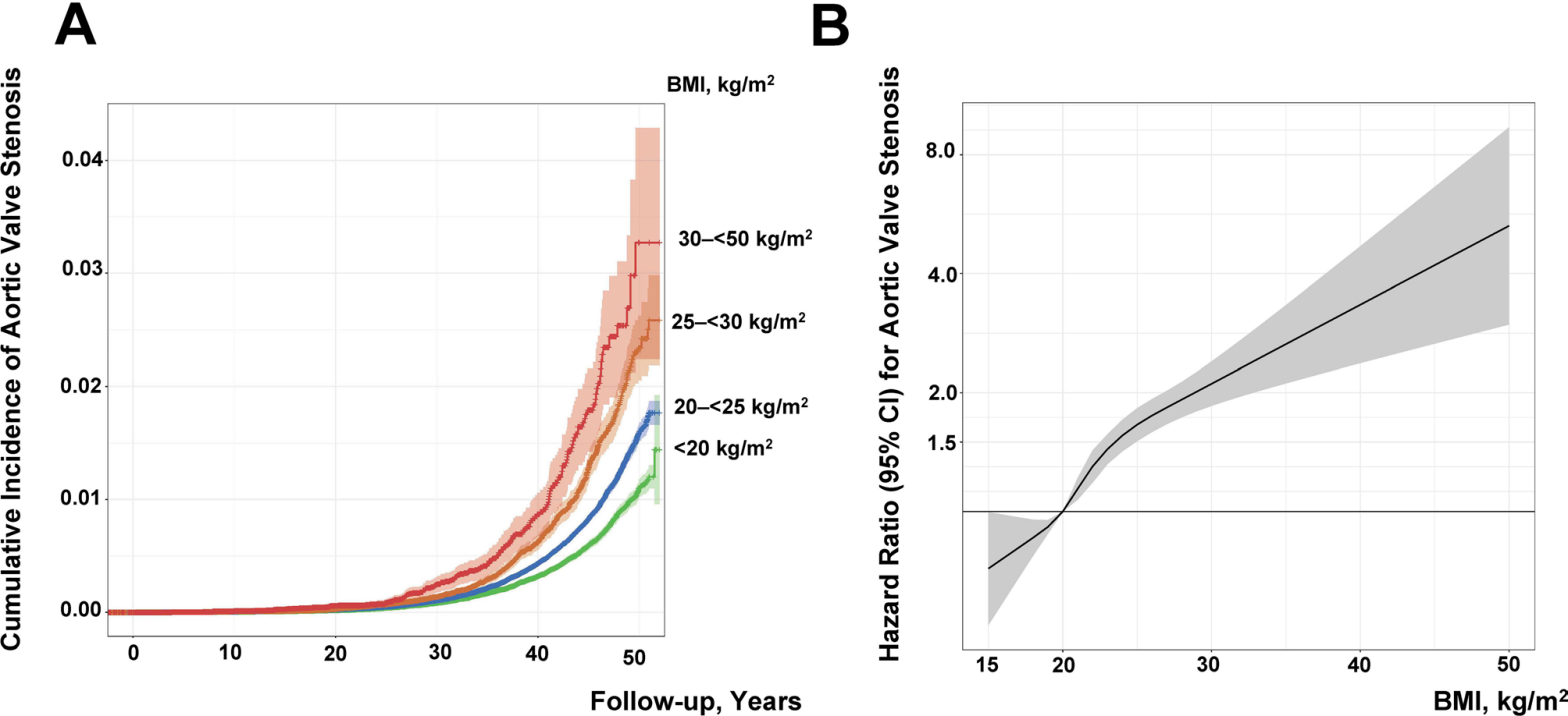
Cumulative incidence plot for aortic valve stenosis according to to body mass index (BMI) group (**A**) and association between BMI at conscription into military service and risk of aortic valve stenosis (**B**). The model was adjusted for age; conscription year (modelled as a spline with knots at 5%, 25%, 75%, and 95% [i.e., 1972, 1979, 1994, and 2002]); test centre; systolic blood pressure; diastolic blood pressure; cardiorespiratory fitness; muscle strength; parental education, and baseline comorbidities (diabetes mellitus, hypertension, congenital heart disease) (*n* = 1 295 253). BMI was restricted to 15 to 50 kg/m^2^ and modelled as a restricted cubic spline with knots at 5%, 25%, 75%, and 95% (i.e., 18.1, 19.9, 23.2, and 27.2 kg/m^2^), with a BMI of 22 kg/m^2^ as the reference.

After a median follow-up of 32 years, 5766 of 1,701,390 (0.34%) men were diagnosed with AVS (Fig. 1, Table S2), with a cumulative incidence of  1.5%  after a maximum follow-up of 50 years. The number of AVS cases in the total cohort was 10.7 per 100 000 person-years, increasing from 9.3 per 100 000 person-years in subjects with BMI of 18.5–<20 kg/m^2^ to 15.8 per 100 000 person-years in the BMI group 27.5–<30 kg/m^2^, with similar event rates in the higher BMI ranges of 30–<35 kg/m^2^ and 35–50 kg/m^2^ (15.0 and 13.9 per 100 000 person-years), respectively. Figure 2A shows the incidence of AVS according to four BMI categories, with an incidence of 3.3% in individuals with obesity, 2.3% in overweight, and 1.6% in those with BMI 20.0–<25.0 kg/m^2^, compared with 1.1% in men with BMI < 20.0 kg/m^2^.

The risk of AVS was 30% lower in men with BMI < 18.5 kg/m^2^ and 20% lower in those with BMI of 18.5–<20 kg/m^2^, compared with those with BMI 20.0–<22.5 kg/m^2^ (Table 2), after multivariable adjustment. Compared with BMI 20.0–<22.5 kg/m^2^, the risk of AVS increased gradually, starting at high-normal BMI levels of 22.5–<25 kg/m^2^ (Table 2; Fig. 2B). Hazard ratios were largely similar in models adjusted for potential confounders including age at conscription, conscription year, test centre, systolic and diastolic blood pressure, IQ, cardiorespiratory fitness, muscle strength, parental education, and baseline comorbidities (diabetes mellitus, hypertension, adult congenital heart disease). The near-linearly increased risk of AVS by BMI category was also reflected as an 8.1% higher risk of AVS (HR, 1.081, 95% CI, 1.069–1.092) for each 1 kg/m^2^ increase in BMI (Table 2).

**Table 2:** Hazard ratios (95% CI) for aortic valve stenosis relative to body mass index group at conscription.

	Model 1*	Model 2†	Model 3‡	Model 4#
Events/population, *n*	5766/1,701,390	5712/1,611,844	3837/1,322,154	3810/1,295,664
BMI < 18.5 kg/m^2^	0.75(0.67–0.83)	0.75(0.68–0.83)	0.78(0.69–0.89)	0.70(0.61–0.80)
BMI 18.5–<20.0 kg/m^2^	0.81(0.75–0.87)	0.82(0.76–0.88)	0.84(0.77–0.92)	0.80(0.73–0.88)
BMI 20.0–<22.5 kg/m^2^	1.00	1.00	1.00	1.00
BMI 22.5–<25.0 kg/m^2^	1.27(1.19–1.36)	1.26(1.17–1.35)	1.25(1.15–1.33)	1.28(1.17–1.39)
BMI 25.0–<27.5 kg/m^2^	1.43(1.29–1.58)	1.38(1.24–1.53)	1.36(1.21–1.55)	1.37(1.21–1.56)
BMI 27.5–<30.0 kg/m^2^	2.01(1.74–2.32)	1.91(1.65–2.21)	1.86(1.55–2.23)	1.78(1.48–2.15)
BMI 30.0–<35.0 kg/m^2^	2.14(1.79–2.56)	1.95(1.63–2.35)	2.10(1.69–2.61)	2.02(1.61–2.52)
BMI 35.0–50 kg/m^2^	2.54(1.72–3.74)	2.35(1.58–3.49)	2.37(1.46–3.83)	1.91(1.12–3.25)
Per unit BMI	1.084(1.076–1.093)	1.079(1.070–1.087)	1.077(1.066–1.088)	1.081(1.069–1.092)

* Model 1 adjusted for age at conscription, conscription year, test centre, and baseline comorbidities (diabetes mellitus, hypertension, and adult congenital heart disease).

†Model 2 as model 1 additionally adjusted for systolic and diastolic blood pressure at baseline.

‡Model 3 as model 2 additionally adjusted for parental education.

#Model 4 as model 3 additionally adjusted for intelligence quotient, cardiorespiratory fitness, and muscle strength.

In the multivariable adjusted models, age, year, diastolic blood pressure, cardiorespiratory fitness, parental education, IQ, and baseline comorbidities such as diabetes mellitus and adult congenital heart disease were independent predictors of AVS (Table S3). There were significant interactions between BMI and diastolic pressure (Table S3).

### Event rates and risk for clinical outcomes in subjects with aortic valve stenosis

During a median follow-up of ~ 5.5 years among patients diagnosed with AVS (*n* = 5766), 555 patients died, including 294 from a cardiovascular cause (Table S4), representing 15.1 and 8.0 events per 1000 person-years for all-cause mortality and cardiovascular mortality, respectively. The event rates of all-cause mortality and cardiovascular mortality increased from 14.0 and 7.1 events per 1000 person-years, respectively, in subjects with BMI < 20 kg/m^2^ to 40.9 and 24.6 per 1000 person-years in those with BMI 30–50 kg/m^2^.

During follow-up of subjects with AVS, 487 developed HF, 947 atrial fibrillation, 212 AMI, and 197 IS (Fig. 1, Table S4). The event rates per 1000 person-years were 15.0 for HF, 31.9 for atrial fibrillation, 6.6 for AMI, and 4.5 for IS among individuals with AVS. In subjects with BMI < 20 kg/m^2^, the event rates per 1000 person-years were HF 12.5, atrial fibrillation 29.6, AMI 4.7, and IS 5.2 compared with event rates of 33.4, 44.8, 7.3, and 8.5, respectively, in those with BMI 30–50 kg/m^2^.

**Fig. 3:**
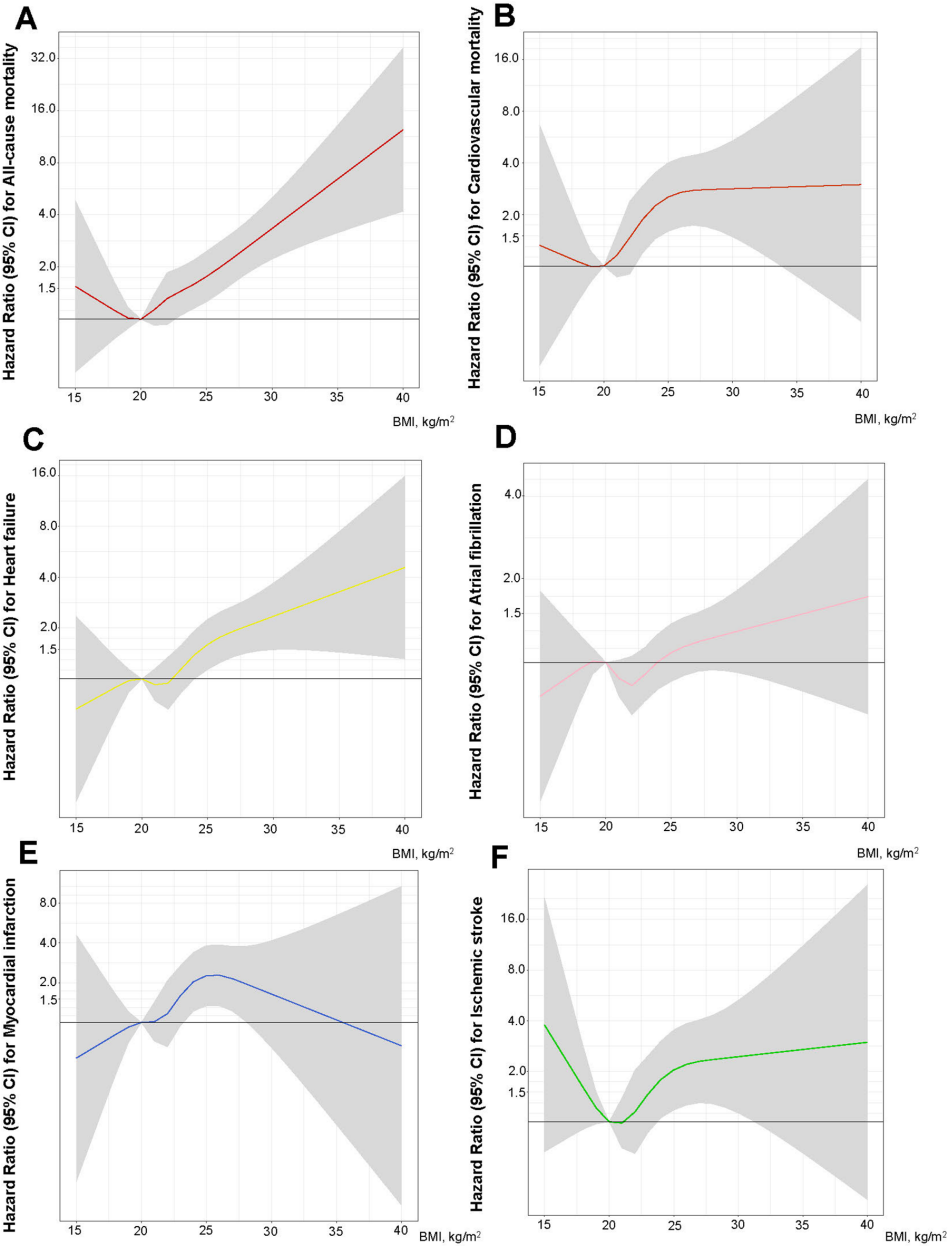
Association between body mass index at conscription and the risk of mortality and cardiovascular outcomes in patients with aortic valve stenosis. **A** Risk of all-cause mortality, **B** cardiovascular mortality, **C** heart failure, **D** Atrial fibrillation/atrial flutter, **E** acute myocardial infarct, and **F** Ischemic stroke in aortic valve stenosis patients according to body mass index. The models were adjusted for age;, conscription year; (modelled as a spline with knots at 5%, 25%, 75%, and 95%[i.e., 1970, 1972, 1982, and 1992]);, test centre; systolic blood pressure; diastolic blood pressure; cardiorespiratory fitness; muscle strength parental education; and baseline comorbidities (diabetes mellitus, hypertension, congenital heart disease). Body mass index (BMI) was restricted to between 15 and 40 kg/m^2^ and modelled as a restricted cubic spline with knots at 5%, 25%, 75%, and 95% (i.e., 18.1, 20.0, 23.5, and 28.0 kg/m)^2^ with a BMI of 20 kg/m^2^ as the reference.

The multivariable-adjusted HR for all-cause mortality and cardiovascular mortality were 4.69 (95% CI, 2.61–8.44) and 4.09 (95% CI, 1.72–9.73), respectively, in men with BMI 30–50 kg/m^2^ compared with those with BMI < 20.0 kg/m^2^ (Fig. 3; Table 3). This was also reflected in the HRs for each 1 kg/m^2^ increase in BMI, which were 11% and 11.5% higher for all-cause mortality and cardiovascular mortality, respectively (Table 3). Similarly, subjects with BMI 30–50 kg/m^2^ had a significantly higher multivariable-adjusted HR for heart failure (HR 2.76, 95% CI, 1.36–5.60) compared with subjects with BMI 20.0 kg/m^2^. In contrast, the associations for BMI and atrial fibrillation and BMI and IS were not statistically significant (Fig. 3). However, it should be noted that the number of atrial fibrillation and IS cases was lower, although the HRs showed similar directional trends.

**Table 3:** Hazard ratios (95% CIs) for subsequent clinical outcomes in patients with aortic valve stenosis relative to body mass index group at conscription.

		Model 1*	Model 2†	Model 3‡	Model 4#
All-cause mortality					
Events/population, n	555/5695		552/5641	313/3793	313/3764
BMI < 20.0 kg/m^2^	1.00		1.00	1.00	1.00
BMI 20.0–<25.0 kg/m^2^	1.06(0.86–1.30)		1.05(0.86–1.29)	1.20(0.91–1.58)	1.35(1.00–1.81)
BMI 25.0–<30.0 kg/m^2^	1.49(1.12–2.00)		1.48(1.10–1.98)	1.74(1.18–2.58)	1.91(1.26–2.90)
BMI 30–<50 kg/m^2^	3.90(2.60–5.84)		3.93(2.61–5.93)	4.31(2.44–7.61)	4.69(2.61–8.44)
Per unit BMI	1.085(1.058–1.112)		1.085(1.058–1.113)	1.098(1.061–1.136)	1.110(1.070–1.115)
Cardiovascular mortality					
Events/population, n	294/5695		293/5641	163/3793	163/3764
BMI < 20.0 kg/m^2^	1.00		1.00	1.00	1.00
BMI 20.0–<25.0 kg/m^2^	1.16(0.87–1.55)		1.14(0.85–1.52)	1.24(0.83–1.84)	1.55(1.02–2.36)
BMI 25.0–<30.0 kg/m^2^	1.79(1.21–2.65)		1.70(1.14–2.53)	1.88(1.10–3.20)	2.34(1.32–4.12)
BMI 30–50 kg/m^2^	5.03(2.96–8.54)		4.59(2.67–7.90)	3.42(1.47–7.92)	4.09(1.72–9.73)
Per unit BMI	1.106(1.070–1.143)		1.100(1.063–1.138)	1.095(1.045–1.148)	1.115(1.063–1.171)
Heart failure					
Events/population, n	487/5206		482/5156	292/3485	290/3458
BMI < 20.0 kg/m^2^	1.00		1.00	1.00	1.00
BMI 20.0–<25.0 kg/m^2^	1.18(0.94–1.47)		1.18(0.94–1.48)	1.09(0.82–1.45)	1.12(0.82–1.53)
BMI 25.0–<30.0 kg/m^2^	1.94 (1.44–2.62)		1.94 (1.44–2.63)	1.94 (1.32–2.87)	2.08 (1.37–3.14)
BMI 30–50 kg/m^2^	2.87(1.71–4.82)		2.61(1.49–4.55)	2.60(1.34–5.04)	2.76(1.36–5.60)
Per unit BMI	1.083(1.054–1.112)		1.079(1.049–1.110)	1.081(1.044–1.120)	1.093(1.052–1.140)
Atrial fibrillation					
Events/population, n	947/5135		939/5086	597/3433	595/3405
BMI < 20.0 kg/m^2^	1.00		1.00	1.00	1.00
BMI 20.0–<25.0 kg/m^2^	1.05(0.90–1.23)		1.06(0.91–1.24)	1.08(0.89–1.32)	0.94(0.76–1.17)
BMI 25.0–<30.0 kg/m^2^	1.49(1.20–1.86)		1.47(1.18–1.84)	1.52(1.15–2.02)	1.27(0.94–1.72)
BMI 30–50 kg/m^2^	1.74(1.14–2.64)		1.72(1.12–2.65)	1.98(1.18–3.33)	1.51 (0.87–2.64)
Per unit BMI	1.043(1.023–1.064)		1.044(1.022–1.066)	1.051(1.024–1.079)	1.031(1.001–1.062)
Myocardial infarction					
Events/population, n	212/5288		210/5234	120/3554	120/3525
BMI < 20.0 kg/m^2^	1.00		1.00	1.00	1.00
BMI 20.0–<25.0 kg/m^2^	1.39(0.98–1.98)		1.41(0.99–2.01)	1.30(0.82–2.04)	0.79(0.51–1.23)
BMI 25.0–<30.0 kg/m^2^	2.55(1.63–3.99)		2.53(1.60–3.99)	2.20(1.21–4.03)	1.85(1.13–3.06)
BMI 30–50 kg/m^2^	2.15(0.84–5.52)		1.73(0.61–4.92)	0.76 (0.10–5.74)	0.49(0.07–3.61)
Per unit BMI	1.102(1.060–1.150)		1.096(1.053–1.141)	1.101(1.041–1.165)	1.116(1.050–1.185)
Ischemic stroke					
Events/population, n	168/5529		168/5476	114/3693	132/3841
BMI < 20.0 kg/m^2^	1.00		1.00	1.00	1.00
BMI 20.0–<25.0 kg/m^2^	0.89 (0.62–1.26)		0.86 (0.61–1.25)	0.96 (0.62–1.49)	0.85 (0.53–1.36)
BMI 25.0–<30.0 kg/m^2^	1.11(0.65–1.90)		1.05(0.61–1.81)	1.20(0.62–2.34)	1.11(0.55–2.24)
BMI 30–50 kg/m^2^	2.24(0.95–5.30)		2.21(0.89–5.08)	2.36(0.81–6.92)	1.90(0.55–6.55)
Per unit BMI	1.041(0.992–1.094)		1.063(0.985–1.089)	1.078(1.019–1.142)	1.074(1.006–1.146)

* Model 1 adjusted for age at conscription, conscription year, test centre, and baseline comorbidities diabetes mellitus, hypertension, and adult congenital heart disease.

†Model 2 as model 1 additionally adjusted for systolic and diastolic blood pressure at baseline.

‡Model 3 as model 2 additionally adjusted for parental education.

#Model 4 as model 3 additionally adjusted for intelligence quotient, cardiorespiratory fitness, and muscle strength.

## Discussion

In this large nationwide long-term prospective study of young men, elevated BMI in adolescence was associated with the development of AVS in adulthood. The elevated risk of AVS in young men was discernible already at high-normal BMI levels (22.5–25 kg/m^2^) and increased incrementally with rising BMI. The linear trend extended down to the underweight range, and adolescents with BMI < 18.5 and 18.5–<20 kg/m^2^ both had a lower risk of developing AVS in early adulthood than the reference low-normal BMI group. Once AVS had developed, further increases in the risk were observed for all-cause mortality, cardiovascular mortality, and heart failure, which were all significantly higher among individuals who were overweight or obese in adolescence compared with lean individuals. This suggests that adiposity in youth may be an indicator of a worse prognosis in established AVS.

High diastolic blood pressure, low cardiorespiratory fitness, low IQ, low parental education, and underlying conditions such as diabetes mellitus and congenital heart disease in adolescence were additional factors associated with an increased occurrence of AVS in adulthood. However, BMI in youth was independently associated with the development of AVS in early adulthood, essentially withstanding adjustment for these factors and comorbidities.

Importantly, overweight and obesity in youth were also associated with a worse prognosis in patients diagnosed with AVS. In particular, young men with obesity who were later diagnosed with AVS in adulthood had elevated risk of all-cause mortality, cardiovascular mortality, and HF after a recorded diagnosis, whereas associations with atrial fibrillation, AMI, and IS were not statistically significant. This may have been due to limited statistical power (fewer cases), but it may also reflect a different pattern of association;  for example, for IS, the relationship appeared more U-shaped, as suggested by the cubic spline modelling.

Only a handful of studies have investigated the long-term impact of risk factors,particularly obesity, on AVS. In the mid-1990s, a cross-sectional imaging study of older adults (median age over 80 years) in the Helsinki Aging Study showed that lower BMI was associated with a higher risk of AVS, potentially because more normal-weight persons survive to older age^20^. Other cross-sectional studies have suggested a neutral or positive association between BMI and AVS^12,21–23^. However, in the prospective Malmö Diet and Cancer Study of 5079 randomly selected participants aged 45–68 years with a 20-year follow-up, each standard deviation (SD) increment of BMI was significantly associated with incident AVS (HR 1.28)^24^, although only a few cases (*n *= 69) of AVS were identified. It has been suggested that obesity is not related to progression of AVS^25^, and, in patients with AVS, lower BMI has been associated with increased all-cause and cardiovascular mortality^26,27^. This may also reflect the severity of the final stages of AVS, when advanced heart failure symptoms are often associated with malnutrition^28^.

To the best of our knowledge, most of the currently available data on the relationship between BMI and AVS development are derived from the Cohort of Swedish Men and the Swedish Mammography Cohort, in which 1297 cases of AVS were identified in 71 817 middle-aged subjects during a follow-up of approximately 15 years^14^. In this study, overweight and obesity were associated with a multivariable-adjusted HRs of 1.24 (95% CI, 1.05–1.48) and 1.81 (95% CI, 1.47–2.23), respectively, for AVS, compared with BMI 18.5–22.4 kg/m^2^. Stratified analyses for BMI < 18.5 kg/m^2^ and for various subclasses of obesity were not performed. Subjects with high-normal BMI (22.5–25 kg/m^2^) and moderate overweight (25–27.5 kg/m^2^) did not have a significantly increased risk of developing AVS, most likely due to limited statistical power, as indicated by non-significant increases in HRs. In the present prospective study of about 1.7 million young men, representative of the Swedish population, with a median follow-up of 32 years and 5766 cases of AVS identified, we observed an essentially linear association between BMI and risk of AVS, with a detectable increase in risk even at very low BMI levels.

The precise mechanisms by which elevated body weight might lead to the development of AVS are, however, unclear. Obese individuals may be predisposed to AVS through hemodynamic conditions such as increased blood volume and cardiac output, which can lead to geometric alterations of the left ventricle and aortic valve^29^. Other potential mechanisms include the effects of obesity on blood pressure and lipid metabolism, as well as overactivity of adipocyte signalling molecules, such as, inflammatory cytokines and oxidative stress, resulting in endothelial dysfunction, and ultimately atherosclerosis, aortic valve fibrosis, and calcification^30,31^. We were not able to adjust for these factors in the follow-up of our study.

Individuals with AVS are at an elevated risk of heart failure and mortality^32,33^, with the adjusted 5-year risk of mortality ranging from mild AVS to severe AVS, reaching up to 3.1-fold in severe cases. However, research investigating the impact of adiposity in youth on cardiovascular outcomes and mortality in patients with established AVS is scarce. Therefore, our results showing a 3- to 5-fold higher risk of all-cause mortality, cardiovascular mortality, and heart failure in men with AVS who were obese when youth compared with normal weight peers, represent a novel finding. Currently, management and follow-up decisions are largely guided by the severity of AVS,  as determined by Vmax, aortic valve gradient, and estimated aortic valve area. Our findings are novel, raise new questions regarding potential mechanisms, and suggest that patients with AVS and a history of obesity in youth may benefit from more vigilant monitoring.

### Strengths and limitations

Strengths of the present study include its large sample size with a high number of AVS cases, long-term follow-up, and a population-based design comprising a cohort largely representative of the male population in Sweden.

There are several limitations to this study. First, the diagnosis of AVS was not formally validated; however, given the relatively high risk of adverse outcomes in individuals with AVS and the fact that ultrasound examinations are a prerequisite for diagnosis in Sweden, we believe the diagnoses were accurate. Second, we used BMI in youth as an indicator of overweight and obesity, with no data on subsequent weight development. BMI trajectories over time could not be assessed, and the findings should therefore be interpreted as reflecting the association between BMI in youth and long-term risk of AVS. Nevertheless, high body weight tends to track over life, and elevated BMI at age 18 is likely a reasonable marker of future risk of overweight and obesity^34^. Although mildly elevated BMI in youth may reflect high muscle mass rather than adiposity this is unlikely when BMI is very high. Other anthropometric measures, such as waist circumference or waist-to-hip ratio were not available. The study population was homogeneous with respect to age, sex, and ethnicity, which limits the generalizability to women and to other populations. While the dataset does not specify the proportion of individuals with bicuspid aortic valve, the cohort included individuals with adult congenital heart disease, which encompasses bicuspid aortic valve. In addition, we were unable to include potentially important factors such as weight changes, smoking, diet, occupation, alcohol consumption, and lipid levels during follow-up. Consequently, the influence of subsequent weight or lifestyle changes on AVS risk cannot be determined. Finally, the register-based design means that AVS reflects a recorded diagnosis rather than undiagnosed disease. Because AVS may remain asymptomatic for many years, differential healthcare utilization could introduce selection bias, particularly if individuals with obesity are more likely to seek care due to dyspnea. Importantly, the observed dose–response relationship between BMI and AVS, including increased risk at high-normal BMI, supports the robustness of our findings and is consistent with a potential causal association, although causality cannot be established. Further studies with repeated BMI and echocardiographic measurements are warranted.

## Conclusions

Mildly elevated BMI in youth is associated with a higher risk of developing AVS later in life, with markedly increased risk among individuals with obesity. Furthermore, excess BMI in youth, when combined with manifest AVS, was associated with a higher risk of subsequent severe clinical outcomes.

## Supplementary Information

Below is the link to the electronic supplementary material.

Supplementary Material 1

## Data Availability

The datasets used in this study contain sensitive personal information from national registries and cannot be made publicly available under Swedish data protection regulations. However, anonymized data may be available from the corresponding author upon reasonable request.
