# Supplementary material for: Impact of body mass index on aortic valve stenosis and its clinical outcomes in early adulthood: a long-term study of young men
Source: Sci Rep. 2026 Mar 26;16:10492. doi: 10.1038/s41598-026-44041-2 (PMC13031306; doi:10.1038/s41598-026-44041-2)
Supplement: Supplementary file 1 — Supplementary Material 1 [file 41598_2026_44041_MOESM1_ESM.docx]

Table S1. ICD codes of aortic valve stenosis and clinical outcomes

| **Diagnosis** | **ICD 8** | **ICD 9** | **ICD 10** |
| --- | --- | --- | --- |
| Aortic valve stenosis | NA | NA | I35.0, I35.2 |
| Heart failure | NA | NA | I50.0–I50.9 |
| Atrial fibrillation/atrial flutter | NA | NA | I48.0–I48.9 |
| Acute myocardial infarct | NA | NA | I21.9 |
| Ischemic stroke | NA | NA | I63.0–I63.9 |
| Diabetes | 250 | 250 | E10–E14 |
| Hypertension | 401–405 | 401–405 | I10–I15 |
| GUCH | 746–747 | 745–747 | Q2, Q332, Q871E, Q872, Q874, Q878, Q90, Q96 |

**Table S2. Event rates per 100 000 person-years for aortic valve stenosis relative to body mass index group**

|  | **All** | **BMI**  **<18.5**  **kg/m^2^** | **BMI**  **18.5–<20.0 kg/m^2^** | **BMI**  **20.0–<22.5 kg/m^2^** | **BMI**  **22.5–<25.0 kg/m^2^** | **BMI 25.0–<27.5 kg/m^2^** | **BMI 27.5–<30.0 kg/m^2^** | **BMI 30.0–35.0 kg/m^2^** | **BMI 35.0–50 kg/m^2^** |
| --- | --- | --- | --- | --- | --- | --- | --- | --- | --- |
| Men, n (% of total) | 1701390  (100) | 138550 (8.1) | 306878 (18.0) | 686154  (40.3) | 361514 (21.2) | 125662 (7.4) | 44399 (2.6) | 30701 (1.8) | 7532 (0.4) |
| Events, n (%) | 5766  (100) | 437  (7.6) | 948  (16.4) | 2285  (39.6) | 1298  (22.3) | 446  (7.7) | 200  (3.5) | 126  (2.2) | 26  (0.5) |
| Cases per 100 000 observed person years (CI) | 10.7  (10.4–11.0) | 9.3  (8.4–10.2) | 9.3  (8.7–9.9) | 10.4  (10.0–10.8) | 11.8  (11.2–12.5) | 12.1  (11.0–13.3) | 15.8  (13.7–18.2) | 15.0  (12.5–17.9) | 13.9  (9.1–20.4) |
| Age at diagnosis, y (SD) | 54.7  (9.4) | 55.7  (9.0) | 55.0  (9.4) | 54.8  (9.4) | 54.4  (10.8) | 54.1  (9.5) | 53.1  (9.5) | 52.6  (10.2) | 48.0  (9.5) |
| Median follow up time, y (IQR) | 32  (24–41) | 35  (26–45) | 34  (26–44) | 33  (24–41) | 31  (22–39) | 29  (21–38) | 28  (20–37) | 26  (19–35) | 24  (18–32) |
| Person-years of follow-up | 53860840 | 4714614 | 10219022 | 21952044 | 10999726 | 3685501 | 1265165 | 837815 | 186955 |

Table S3. Hazard Ratios (95% CIs) for Aortic Valve Stenosis by cardiovascular risk factors

|  | Multivariable adjusted Model |
| --- | --- |
| Events/population, n | 3810/1295664 |
| **Age** | 1.06(1.00–1.11) |
| P-value interaction | 0.6 |
| **Center** | 0.99(0.97–1.01) |
| P-value interaction | 0.7 |
| **Year** | 1.02(1.02–1.03) |
| P-value interaction | 0.1 |
| **Systolic blood pressure** |  |
| 100–119 mmHg | 1 |
| 120–125 mmHg | 0.95(0.86–1.05) |
| 126–130 mmHg | 0.91(0.82–1.01) |
| 131–138 mmHg | 0.92(0.82–1.02) |
| 139–180 mmHg | 0.97(0.87–1.08) |
| P-value interaction | 0.8 |
| **Diastolic blood pressure** |  |
| 40–59 mmHg | 1 |
| 60–65 mmHg | 1.03(0.91–1.16) |
| 66–70 mmHg | 1.20 (1.07–1.34) |
| 71–76 mmHg | 1.19(1.05–1.34) |
| 77–100 mmHg | 1.49(1.33–1.67) |
| P-value interaction | 0.01 |
| **Cardiorespiratory fitness** |  |
| High (8-9) | 1 |
| Moderate (5-7) | 1.16(1.08–1.25) |
| Low (1-4) | 1.21(1.10–1.33) |
| P-value interaction | 0.9 |
| **Muscle strength** |  |
| High (8-9) | 1 |
| Moderate (5-7) | 1.00(0.93–1.08) |
| Low (1-4) | 1.09(0.96–1.23) |
| P-value interaction | 1.0 |
| **Parental education** |  |
| High (8-9) | 1 |
| Moderate (5-7) | 1.26(1.12–1.41) |
| Low (1-4) | 1.35(1.19–1.53) |
| P-value interaction | 0.5 |
| **IQ** |  |
| High (8-9) | 1 |
| Moderate (5-7) | 1.10(1.01–1.19) |
| Low (1-4) | 1.13(1.02–1.25) |
| P-value interaction | 0.08 |
| **Baseline comorbidities** |  |
| Diabetes | 3.93(1.96–7.87) |
| P-value interaction | 0.9 |
| Hypertension | 1.26(0.69–2.28) |
| P-value interaction | 0.8 |
| Adult congenital heart disease | 37.82(30.25–47.27) |
| P-value interaction | 0.5 |

Missing values: Systolic blood pressure (n=85 720), diastolic blood pressure (n=86 145), parental education (n=293 749), muscle strength (n=12 845), cardiorespiratory fitness (n=44744), and IQ (n=12557).

**Table S4. Event rates per 1000 person-years for clinical outcomes in patients with aortic valve stenosis relative to body mass index group**

|  | **All** | **BMI<20.0**  **kg/m^2^** | **BMI 20.0–<25.0 kg/m^2^** | **BMI 25.0–30.0 kg/m^2^** | **BMI 30–50 kg/m^2^** |
| --- | --- | --- | --- | --- | --- |
| **All-cause mortality** | | | | | |
| Men, n (% of total) | 5695(100) | 1369(24.0) | 3538(62.1) | 639(11.2) | 149(2.6) |
| Events, n (%) | 555(100) | 132(23.8) | 321(57.8) | 72(13.0) | 30(5.4) |
| Cases per 1000 observed person years(CI) | 15.1(13.9–16.5) | 14.0(11.8–16.7) | 14.1(12.6–15.7) | 18.9(14.8–23.8) | 40.9(27.6–58.4) |
| Age at death, y (SD) | 58.8(7.1) | 58.5(7.0) | 59.0(7.30) | 58.6(7.6) | 57.9(8.2) |
| Median follow up time, y (IQR) | 5.6(2.3–11.8) | 5.9(2.4–11.5) | 5.6(2.3–10.6) | 5.4(2.1–10.2) | 4.7(2.0–10.0) |
| Person-years of follow up | 36662 | 9398 | 22720 | 3809 | 733 |
| **Cardiovascular mortality** | | | | | |
| Men, n (% of total) | 5695(100) | 1369(24.0) | 3538(62.1) | 639(11.2) | 149(2.6) |
| Events, n(%) | 294(100) | 64(21.8) | 170(57.8) | 42(14.3) | 18(6.1) |
| Cases per 1000 observed person years (CI) | 8.0 (7.0–8.7) | 7.1(5.2–9.0) | 7.5(6.4–8.7) | 11.0(7.9–14.9) | 24.6(14.6–38.8) |
| Age at CV death, y (SD) | 58.5(7.1) | 58.2(7.1) | 58.9(6.6) | 56.9(8.6) | 59.4(7.9) |
| Median follow up time, y (IQR) | 5.3(2.1–10.3) | 5.6(2.3–11.0) | 5.3(2.2–10.1) | 5.1(2.0–9.6) | 4.1(1.6–8.2) |
| Person-years of follow-up | 36662 | 9398 | 22720 | 3810 | 733 |
| **Heart Failure** | | | | | |
| Men, n (% of total) | 5207(100) | 1255(24.7) | 3268(62.4) | 564(10.5) | 119(2.3) |
| Events, n (%) | 487(100) | 105(23.1) | 289(59.5) | 75(13.6) | 18(3.8) |
| Cases per 1000 observed person years (CI) | 15.0(13.7–16.4) | 12.5(10.2–15.2) | 14.3(12.7–16.0) | 23.3(18.3–29.2) | 32.4(19.2–51.1) |
| Age of diagnosis, y (SD) | 58.1(7.1) | 57.9(7.4) | 58.8(6.8) | 56.3(7.8) | 54.7(7.3) |
| Median follow up time, y (IQR) | 5.3(2.1–10.3) | 5.6 (2.2–11.1) | 5.3 (2.1–10.1) | 5.3 (2.0–9.9) | 4.1 (1.9–7.0) |
| Person-years of follow up | 32380 | 8386 | 20217 | 3219 | 556 |
| **Atrial Fibrillation** | | | | | |
| Men, n (% of total) | 5136(100) | 1242(24.2) | 3195(62.2) | 575(11.2) | 123(2.4) |
| Events, n(%) | 947(100) | 225(23.8) | 568(60.0) | 129(13.6) | 25(2.6) |
| Cases per 1000 observed person years (CI) | 31.9(29.9–34.0) | 29.6(25.9–33.8) | 30.6(28.1–33.2) | 43.6(36.4–51.8) | 44.8(29.0–66.2) |
| Age at diagnosis, y (SD) | 58.2(7.3) | 58.3(6.7) | 58.7(7.1) | 56.9(7.4) | 53.4(10.4) |
| Median follow up time, y (IQR) | 5.5(2.2–10.7) | 5.8 (2.2–11.6) | 5.5 (2.2–10.6) | 5.3 (2.0–10.0) | 4.1(1.9–8.2) |
| Person-years of follow up | 29703 | 7592 | 18591 | 3962 | 558 |
| **Myocardial Infarction** | | | | | |
| Men, n (% of total) | 5288(100) | 1266(23.9) | 3300(62.4) | 587(11.1) | 135(2.6) |
| Events, n(%) | 212(100) | 41(19.3) | 129(60.8) | 37(17.5) | 5(2.4) |
| Cases per 1000 observed person years (CI) | 6.6(5.4–7.1) | 4.7(3.3–6.3) | 6.1(5.1–7.2) | 10.6(7.5–14.7) | 7.3(2.4–16.9) |
| Age at diagnosis, y (SD) | 59.0(6.4) | 59.4(6.3) | 59.4(6.4) | 58.0(5.3) | 54.7(13.0) |
| Median follow up time, y (IQR) | 5.3(2.1–10.2) | 5.6(2.2–11.1) | 5.3(2.2–10.1) | 5.1(2.0–9.5) | 3.6(1.3–7.3) |
| Person-years of follow up | 34251 | 8813 | 21269 | 3479 | 689 |
| **Ischemic Stroke** | | | | | |
| Men, n (% of total) | 5530(100) | 1327(24.6) | 3438(61.9) | 616(10.9) | 148(2.7) |
| Events, n (%) | 197(100) | 47(28.4) | 96(57.9) | 19(10.7) | 6(3.0) |
| Cases per 1000 observed person years (CI) | 4.5(3.9–5.2) | 5.2(3.8–6.9) | 4.4(3.5–5.3) | 5.3(3.2–8.2) | 8.5(3.1–18.5) |
| Age at diagnosis, y (SD) | 58.5(7.1) | 59.7(6.6) | 58.7(6.6) | 56.2(6.8) | 52.4(14.1) |
| Median follow up time, y (IQR) | 5.2(2.1–10.0) | 5.4(2.2–10.8) | 5.2(2.1–10.0) | 4.8(1.9–8.9) | 3.2(1.3–6.7) |
| Person-years of follow up | 35335 | 9018 | 21998 | 3613 | 705 |

**Figure S1. Association between body mass index at conscription and risk for Aortic Valve Stenosis**

The model was unadjusted (n= 1 700 706). BMI was restricted to BMI between 15 and 50 kg/m^2^ and modelled as a restricted cubic spline with knots at 5%, 25%, 75%, and 95% (i.e. 18.1, 19.9, 23.2, and 27.2 kg/m^2^), with BMI of 22 kg/m^2^ as reference.

**Figure S2. Association between body mass index at conscription and risk for all-cause mortality in patients diagnosed with Aortic Valve Stenosis**

The model was unadjusted (n=5693). BMI was restricted to BMI between 15 and 40 kg/m^2^ and modelled as a restricted cubic spline with knots at 5%, 25%, 75%, and 95% (i.e. 18.1, 20.1, 23.5, and 28.1 kg/m^2^), with BMI of 20 kg/m^2^ as reference.

**Figure S3. Association between body mass index at conscription and risk for CVD mortality in patients diagnosed with Aortic Valve Stenosis**

The model was unadjusted (n=5693). BMI was restricted to BMI between 15 and 40 kg/m^2^ and modelled as a restricted cubic spline with knots at 5%, 25%, 75%, and 95% (i.e. 18.1, 20.0, 23.4, and 28.1 kg/m^2^), with BMI of 20 kg/m^2^ as reference.

**Figure S4. Association between body mass index at conscription and risk for HF in patients diagnosed with Aortic Valve Stenosis**

The model was unadjusted (n=5202). BMI was restricted to BMI between 15 and 40 kg/m^2^ and modelled as a restricted cubic spline with knots at 5%, 25%, 75%, and 95% (i.e. 18.0, 20.0, 23.4, and 27.8 kg/m^2^), with BMI of 20 kg/m^2^ as reference.

**Figure S5. Association between body mass index at conscription and risk for AF in patients diagnosed with Aortic Valve Stenosis**

The model was unadjusted (n=5131). BMI was restricted to BMI between 15 and 40 kg/m^2^ and modelled as a restricted cubic spline with knots at 5%, 25%, 75%, and 95% (i.e. 18.0, 20.0, 23.5, and 28.1 kg/m^2^), with BMI of 20 kg/m^2^ as reference.

**Figure S6. Association between body mass index at conscription and risk for AMI in patients diagnosed with Aortic Valve Stenosis**

The model was unadjusted (n=5284). BMI was restricted to BMI between 15 and 40 kg/m^2^ and modelled as a restricted cubic spline with knots at 5%, 25%, 75%, and 95% (i.e. 18.0, 20.0, 23.5, and 28.1 kg/m^2^), with BMI of 20 kg/m^2^ as reference.

**Figure S7. Association between body mass index at conscription and risk for IS in patients diagnosed with Aortic Valve Stenosis**

The model was unadjusted (n=5525). BMI was restricted to BMI between 15 and 40 kg/m^2^ and modelled as a restricted cubic spline with knots at 5%, 25%, 75%, and 95% (i.e. 18.1, 20.0, 23.5, and 28.1 kg/m^2^), with BMI of 20 kg/m^2^ as reference.
